# The Impact of Spring Ligament Injuries on Flatfoot Deformity: An Exploratory Study of Morphological and Radiographic Changes in 198 Patients

**DOI:** 10.3390/jcm14145109

**Published:** 2025-07-18

**Authors:** Roxa Ruiz, Roman Susdorf, Beat Hintermann

**Affiliations:** Center of Excellence Foot and Ankle Surgery, Clinic for Orthopaedics and Traumatology, University of Basel, Kantonsspital Baselland, 4104 Bruderholz, Switzerland; roxa.ruiz@ksbl.ch (R.R.); roman.susdorf@ksbl.ch (R.S.)

**Keywords:** spring ligament, posterior tibial tendon, injuries, flatfoot, progressive collapsing flatfoot deformity, trauma

## Abstract

**Background**: Spring ligament (SL) injuries are primarily associated with progressive collapsing flatfoot deformity, but can also occur due to trauma. It remains unclear whether the morphological changes following trauma differ from those caused by chronic overload. The aim of this study was (1) to analyze whether a relationship exists between the injury pattern and foot deformity and (2) to evaluate whether there is a distinction between trauma-related and non-trauma-related injuries. **Method**: We prospectively enrolled 198 patients with a median age of 57 years (range, 13 to 86 years; female, 127 (64%); male, 71 (36%)) who had a clinically diagnosed, surgically confirmed, and classified SL injury. We used weight-bearing standard X-rays to assess foot deformity. The control group consisted of 30 patients (median age 51 years, range, 44–66; female, 21 (70.0%); male, 9 (30.0%)) with no foot deformities or prior foot surgeries. **Results**: A 41.9% incidence of trauma was identified as the cause of these injuries, accounting for 16 (20.8%) of isolated injuries to the SL, 30 (42.9%) of SL injury accompanied by a posterior tibial (PT) tendon avulsion, and 37 (72.5%) of SL injury alongside a bony avulsion at the navicular injuries. The odds of being post-traumatic decreased with each year of age by a factor of 0.97 (95% CI: 0.95–0.99). **Conclusions**: While all radiographic measurements for flatfoot deformity became pathological after an injury to the SL, they did not accurately predict the injury patterns of the SL and distal PT tendon. Generally, post-traumatic cases exhibited lower severity of foot deformity, suggesting that other structures beyond the SL may contribute to the development of flatfoot deformity.

## 1. Introduction

The deltoid-spring ligament complex—a large confluent ligament consisting of the spring (SL) and deltoid (DL) ligaments, also known as the tibiocalcaneonavicular ligament [1,2,3,4]—has only recently been identified as a crucial player in progressive flatfoot deformities [5,6,7,8,9]. Recent studies emphasize that deltoid-spring ligament pathology is as prevalent or even more significant than an insufficient posterior tibial (PT) tendon [10,11], which has traditionally been seen as the key contributor to progressive flattening of the foot arch [12,13]. Deltoid-spring ligament incompetence may develop due to trauma [14,15] or chronic overload, as observed in patients with a progressive hindfoot valgus deformity [10,16,17,18,19]. There is no evidence in the literature regarding whether the morphological changes to the deltoid-spring ligament after trauma differ from those observed after chronic overload. Furthermore, it remains unknown to what extent the integrity of the PT tendon may also be affected. In addition, radiographic data on morphological changes in the foot and ankle are still sparse, and their value in predicting injuries to the soft tissue remains unclear [10,20,21]. Consequently, the value of diagnostic measures and recommendations for ligament reconstructions and additional procedures remains somewhat arbitrary.

**Figure 1:**
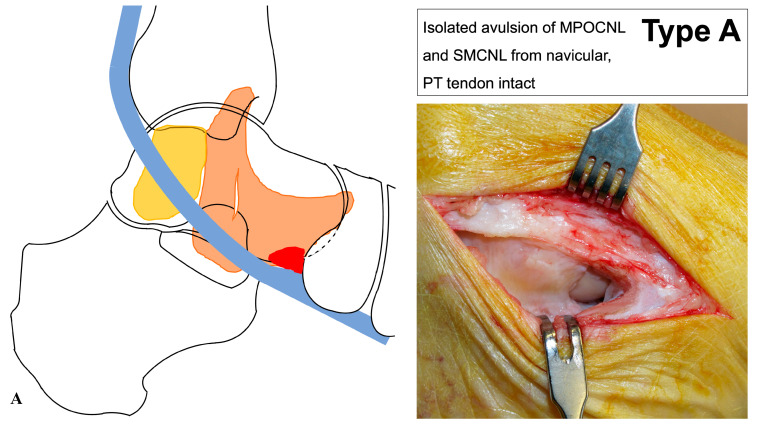

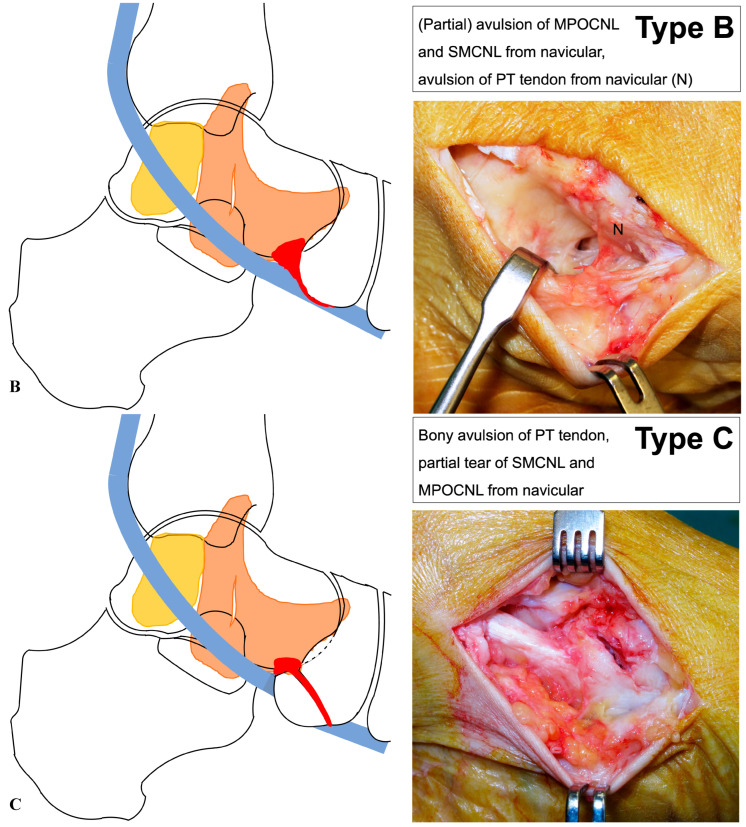
Classification of injury patterns to the deltoid-spring ligament complex [5]. (**A**) Injury Type A: pure ligamentous injuries; (**B**) Injury Type B: ligamentous injuries associated with a distal avulsion of the PT tendon; and (**C**) Injury Type C: ligamentous injuries related to an accessory navicular bone (os tibial externum) that has lost its firm connection to the navicular.

Recently, three distinct types of injuries to the deltoid-spring ligament complex were proposed (Table 1), categorizing injuries to the SL into pure ligamentous injuries (Type A), ligamentous injuries associated with a distal avulsion of the PT tendon (Type B), and ligamentous injuries related to an accessory navicular bone (os tibial externum) that has lost its firm connection to the navicular (Type C) (Figure 1) [5]. The aim of this study was (1) to analyze whether a relationship exists between the injury pattern and foot deformity, and (2) to evaluate whether a distinction can be made between trauma-related and non-trauma-related injuries. We hypothesized that an SL injury causes morphological changes in the foot, which are smaller in post-traumatic cases due to its acute onset.

## 2. Materials and Methods

This study followed the Declaration of Helsinki and the Guidelines for Good Clinical Practice. The Institutional Review Board approved this study (Ethical committee Nordwest- und Zentralschweiz 2021—01972/28 October 2020), and written consent was obtained from each patient. There was no external source of funding. All patients who underwent surgical treatment for an SL injury between January 2016 and December 2020 were prospectively analyzed. Criteria for study inclusion were (1) a clinically diagnosed SL injury that was confirmed during subsequent surgical repair, (2) the presence of preoperative standard weight-bearing radiographs, including an alignment view performed at our institution, and (3) a signed informed consent form. Exclusion criteria were (1) a valgus tilt of the talus indicating an incompetence of deep DL in the mortise view, (2) any previous surgery of the foot and ankle including correcting osteotomy, arthrodesis, ligament reconstruction or tendon transfer, (3) osteoarthritis of any hindfoot and/or midfoot joint or (4) the presence of a coalition and/or a neurological disorder. One hundred and ninety-eight patients with a median age of 57 years (range, 13–86 years; female, 127 [64%]; male, 71 [36%]) were confirmed eligible and analyzed in a prospective pre-post study design before and after surgical repair. A group of 30 patients (median age 51 years [range, 40–66]; female, 21 [70.0%]; male 9 [30.0%]) with no foot deformities or prior foot surgeries was used as a control group.

### 2.1. Clinical Diagnosis

An injury to the SL was suspected when patients presented with a flattening of the arch and persistent pain at the PT tendon insertion area on the navicular and/or beneath the medial arch. This condition could occur with hindfoot valgus and forefoot abduction, either after trauma (typically from a forceful landing on a pronated foot) or developed insidiously without apparent trauma. Patients who reported a sensation of diminished medial foot stability and experienced giving way, especially when fatigued and walking on uneven ground, were also considered to have an injury to the deltoid-spring ligament complex. Generally, the patients could achieve a single heel rise; however, the overall foot deformity only partially resolved. In most cases, the patient could generate a supination-adduction force to the forefoot, indicating preserved function of the PT tendon. The clinical findings and history were systematically recorded.

### 2.2. Imaging Diagnosis

**Figure 2:**
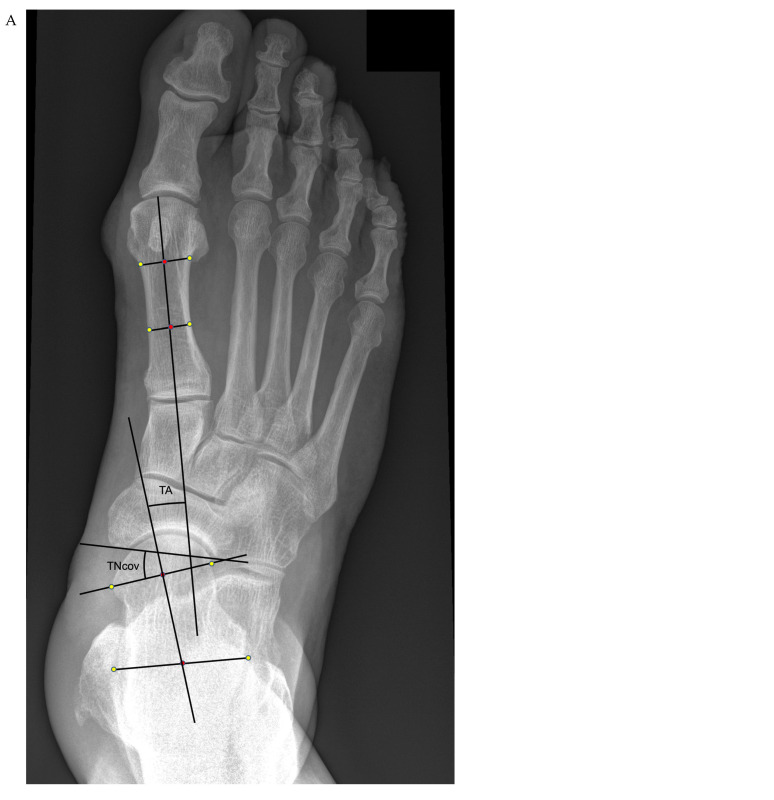

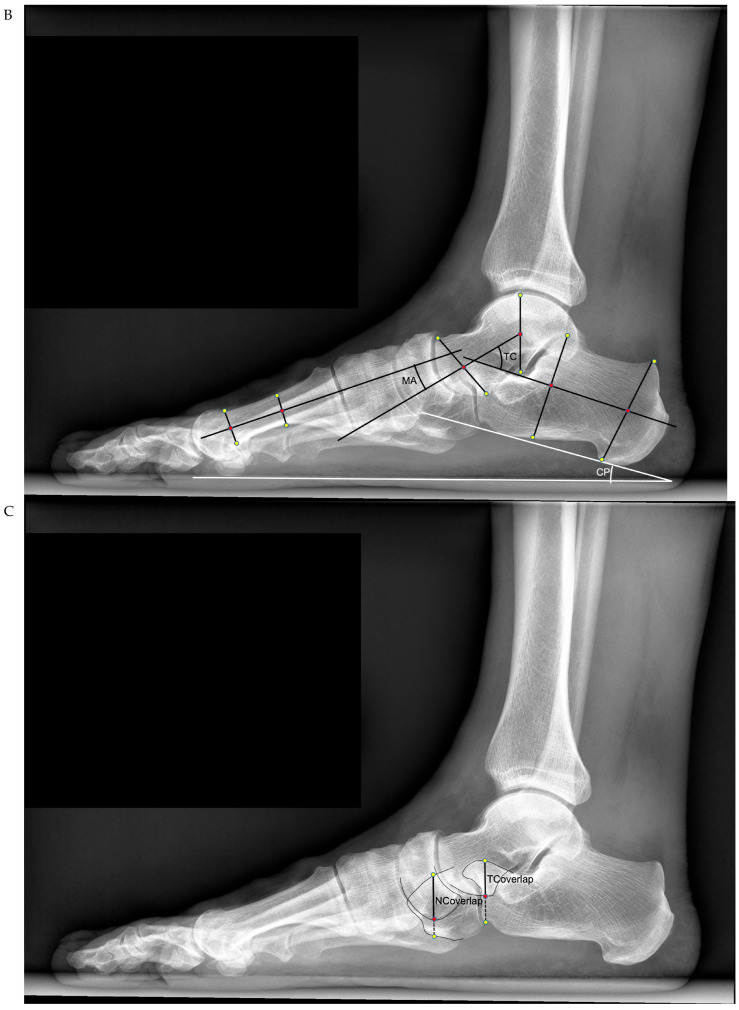

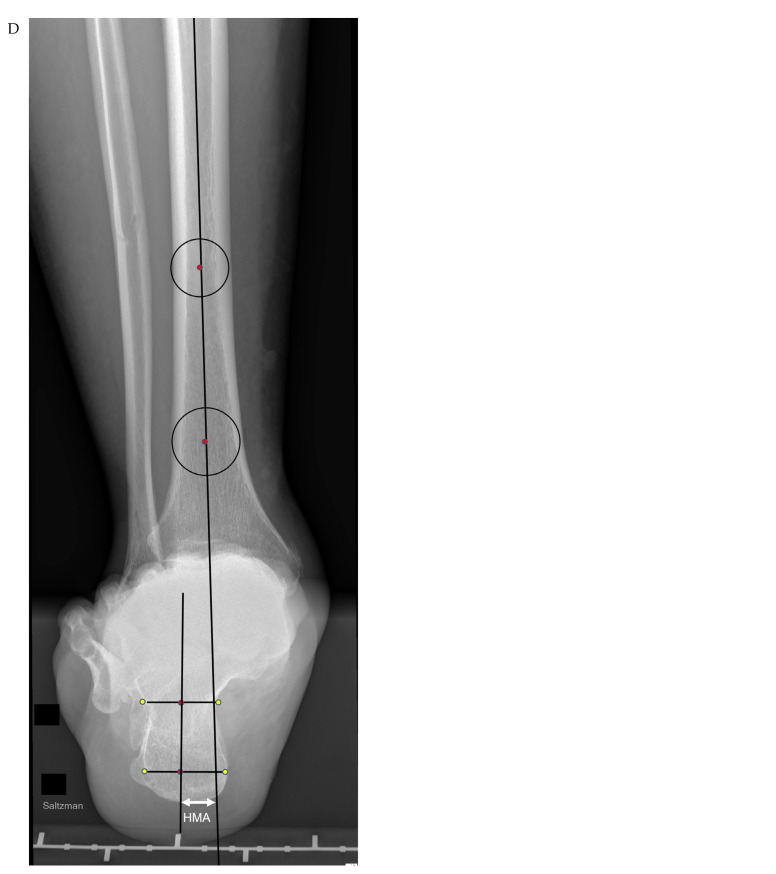
Radiographic measurements. (**A**) DP view: talonavicular coverage (TNcov) angle and talo-first metatarsal (TM) angle; (**B**) lateral view: lateral talo-first metatarsal (Meary’s, MA) angle, calcaneal inclination (Pitch, CP) angle, and lateral talocalcaneal (TC) angle; (**C**) lateral foot view (cont.): talocalcaneal overlap (TCoverlap) and naviculocuboid overlap (NCuboverlap); (**D**) Saltzman alignment view: heel moment arm (HMA).

Preoperative weight-bearing radiographs, including an AP (mortise) view of the ankle, a lateral view, a DP view of the foot, and a hindfoot alignment view [22], were assessed using the SECTRA (Linköping, Sweden) picture archiving and communication system (PACS) by a separate fellowship-trained foot and ankle surgeon who was blinded to the intraoperative findings. Measurements included (1) AP talonavicular coverage (TNcov) angle, (2) talo-first metatarsal (TM) angle, (3) lateral talo-first metatarsal (Meary’s, MA) angle, (4) calcaneal inclination (Pitch, CP) angle, (5) lateral talocalcaneal (TC) angle, (6) heel moment arm (HMA [22]), (7) talocalcaneal overlap (TCoverlap), and (8) naviculocuboid overlap (NCuboverlap) (Table 2, Figure 2). A bony avulsion or an accessory bone at the insertion of the PT tendon to the navicular was also noted. An MRI or a weight-bearing CT scan was not routinely used. Test-/re-test measurements were conducted in 20 randomly selected patients to determine the intra- and inter-observer reliability.

### 2.3. Surgical Exploration

A 3 to 5 cm incision was made along the course of the PT tendon, from the tip of the medial malleolus to the navicular. After dissecting its sheath, the condition of the PT tendon was carefully inspected. If incompetence or rupture of the tendon was found, a lesion in its intratendinous part was separated from an avulsion at its insertion point to the navicular bone. An intratendinous lesion included morphologic changes following degeneration, elongation, and rupture. In cases of a distal lesion, two types of injury were distinguished: an avulsion injury without or with bone involvement (e.g., an accessory bone that has lost its firm connection to the navicular or an acute fracture of the navicular tuberosity). While a retractor held away the tendon, the DL and SL were explored visually and mechanically using forceps. At the same time, the foot was moved from supination-adduction to pronation-abduction and vice versa. If no transmural rupture was visible, but the ligamentous complex appeared incompetent in a forced pronation-abduction position, it was classified as a partial or incomplete rupture of the superficial DL (tibionavicular and/or tibiospring ligament) or SL, accordingly to where the incompetence of the tissue was perceived during visual inspection and/or manual testing. Conversely, a lesion exposing the talar head was classified as a transmural or complete rupture. The findings were documented in a standard format (Table 3), and photographs were taken to capture the soft tissue injury.

According to the classification of Hintermann and Ruiz [5], the injury was classified as Type A, Type B, or Type C injury (Figure 1).

### 2.4. Statistical Analyses

Shapiro–Wilk tests were used to assess the normal distribution of the data. Differences between the injury and control groups were assessed using Wilcoxon rank sum tests for continuous data and Fisher’s exact tests for categorical data. The resultant *p*-values were adjusted using the Benjamini-Hochberg correction.

The analysis was carried out in two parts to avoid overfitting: in the first analysis, we assessed the association between radiological outcomes (Table 1) and predetermined variables related to patient characteristics: (i) Type, (ii) Trauma, (iii) Sex, (iv) Age, as well as the two-way interaction term between (v) Sex and Trauma. In the second analysis, we assessed the association between radiological outcomes (Table 1) and injury patterns: (i) SL lesion severity (partial or complete), (ii) location (distal or intermedial), and (iii) deltoid ligament lesion (presence or absence), controlling for age and trauma.

We applied linear regression (for a continuous response variable) or logistic regression (for a binary response variable) and used model averaging for inference. We started with a global model that included all assessed variables and employed the R function dredge from the MuMIn package to fit a set of candidate models containing all possible variable combinations. For model selection, we used AICc, selecting models from the subset with an AICc difference of ≤2 relative to the lowest AICc in the set. An average model from this subset was derived using conditional (rather than full) averages of coefficients (see MuMIn package vignette for further reference). The 95% confidence intervals (95% CI) were calculated as mean ±1.96 SD (R function confint). We simulated the accuracy of predictions for injury type using multinomial regression with all possible combinations of one or more radiological measurements fitted to a training data subset containing 50–95% of all patients. Using 10,000 repetitions, we compared the model’s predictions on the remaining patients (test data subset) to the actual outcomes. Statistical analysis was conducted using R Project for Statistical Computing (version 3.4.3) via RStudio Server Pro (version 1.4.1103-3).

## 3. Results

The baseline characteristics of the included patients are depicted in Table 4. The patients with a Type C injury were significantly younger than those with a Type A or Type B injury, and the amount of post-traumatic cases was also significantly higher in this group.

**Figure 3:**
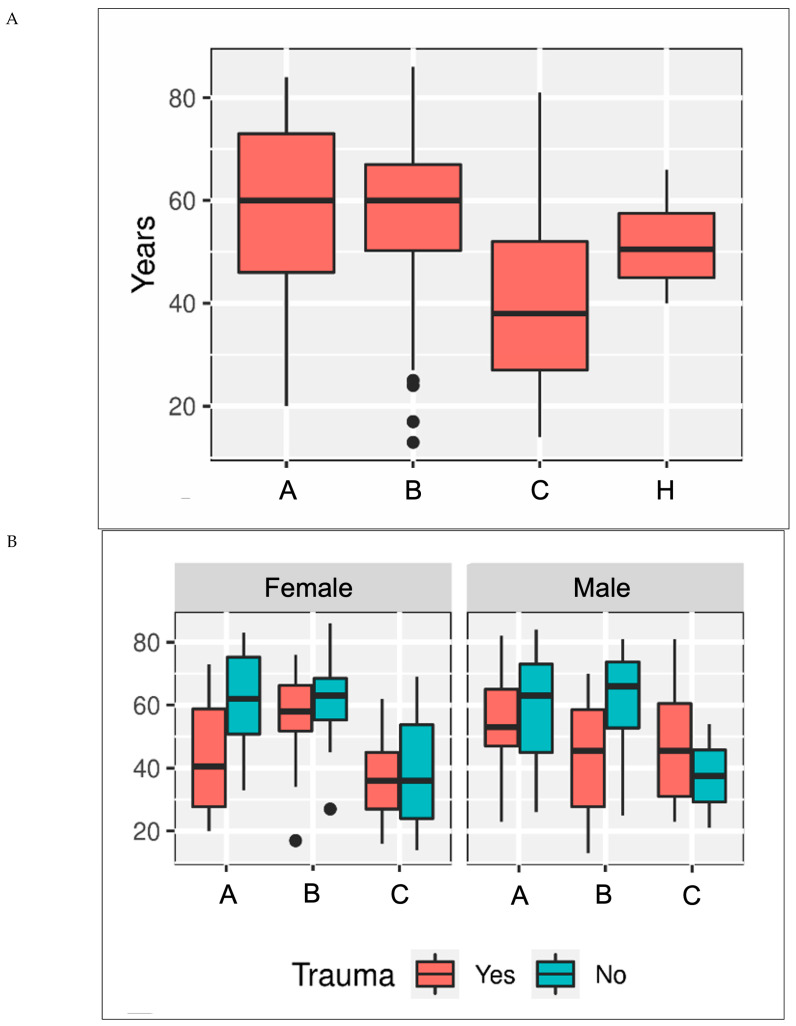

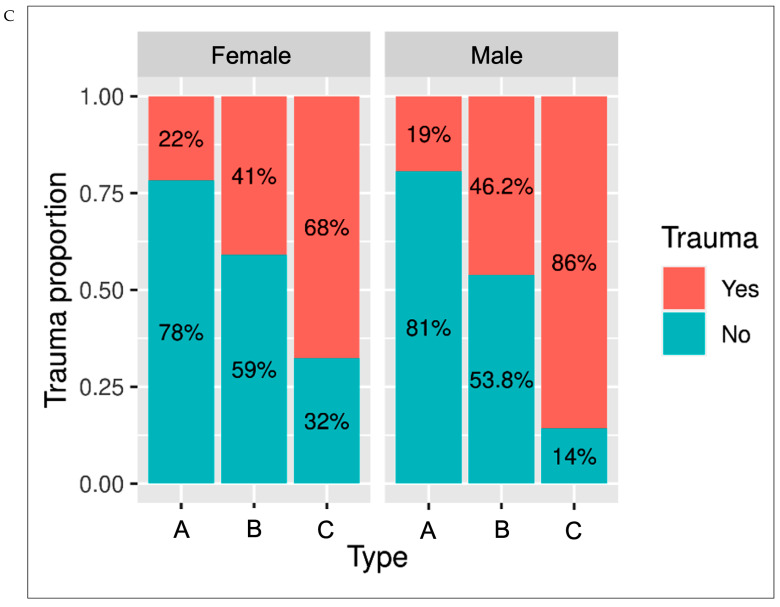
(**A**) Age (in years) of patients with SL injuries (Types A, B, and C) and healthy controls (H); (**B**) age of patients considering sex (F = female, M = male) and trauma as the cause; and (**C**) proportion of trauma cases in patients with SL injuries categorized into Types A, B, and C.

The median age in the injury group was 57 years (range 13–86) and did not differ from the control group (51 years [range 40–66]; *p* = 0.299) (Figure 3A). Patients with a Type C injury were significantly younger (37 years [range 26–52]; *p* < 0.001). Generally, patients with a history of trauma were 8 years (95% CI: 3–13) younger than those without trauma (Figure 3B).

The odds of being post-traumatic declined with each passing year of age by a factor of 0.97 (95% CI: 0.95–0.99). Compared to Type C, the odds ratio for being post-traumatic in Type A and Type B was reduced by 0.15 (95% CI: 0.06–0.35) and 0.43 (95% CI: 0.18–0.99), respectively. The odds ratio for male patients being post-traumatic increased by a factor of 1.2 (95% CI: 0.6–2.4) (Figure 3C).

**Figure 4:**
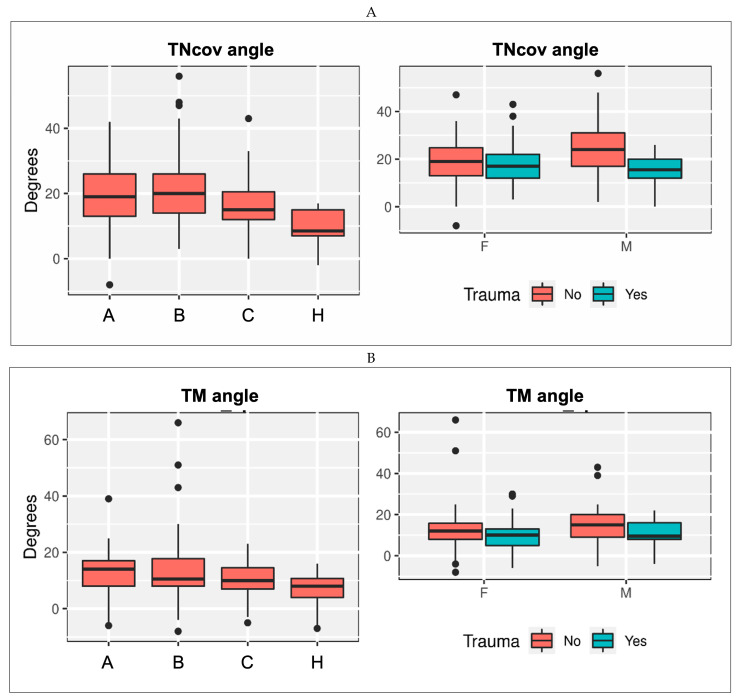

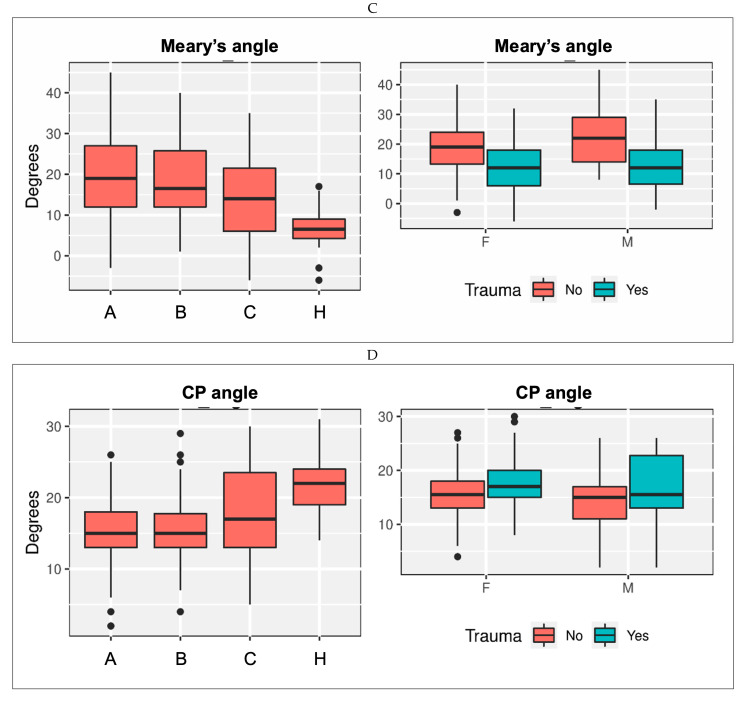

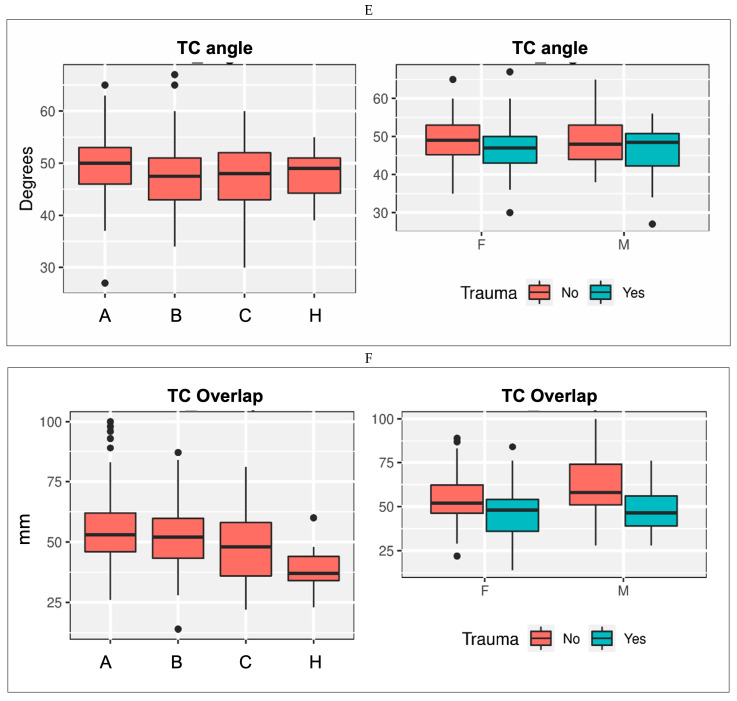

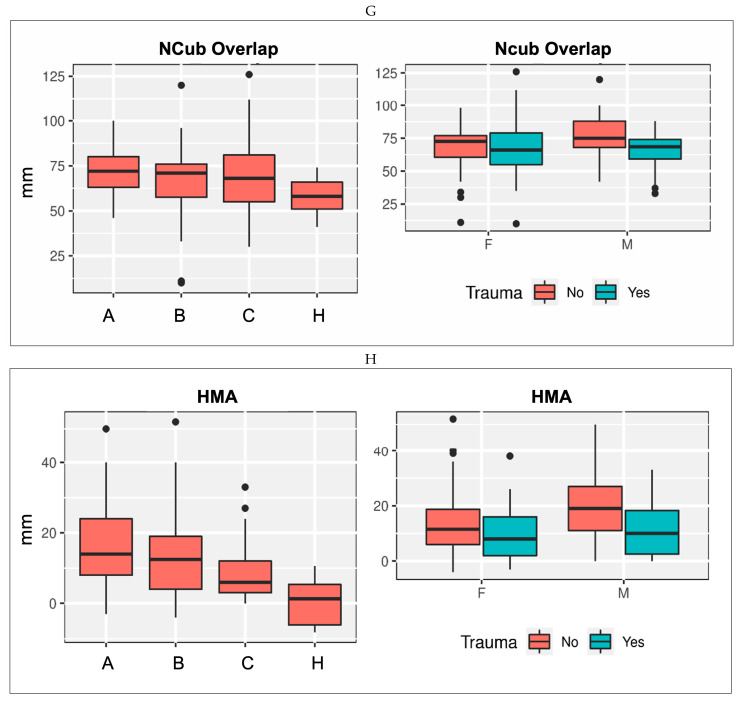
Radiologic measurements in patients with SL injuries (Types A, B, and C) and healthy controls (H) in consideration of gender (F = female, M = male) and trauma as the cause. (**A**) TNcov angle (in degree); (**B**) TM angle (in degree); (**C**) Meary’s angle (in degree); (**D**) Calcaneal inclination (Pitch) angle (in degree); (**E**) TC angle (in degree); (**F**) TCoverlap (in mm); (**G**) TCuboverlap (in mm); (**H**) Heel moment arm (in mm).

Based on the findings from surgical exploration, all patients could be categorized by consent into one of the three injury types (Table 5). Except for the TC angle, a difference between the injury and the control group was found for all radiographic measurements (Table 6, Figure 4). The degree of deformity in injury Types A and B was relatively similar, and overall more significant than in Type C.

**Figure 5:**
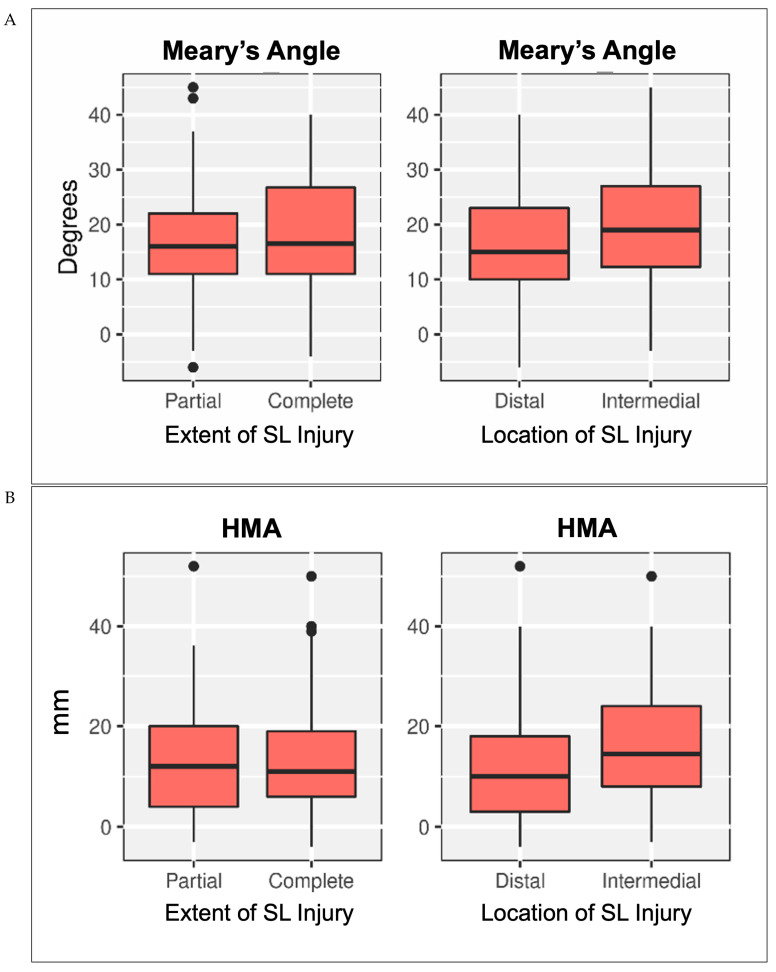
Impact of the extent and location of the SL injury on (**A**) Meary’s angle (in degrees), and (**B**) HMA (in mm).

The extent (partial/complete) and location (intermediate/distal) of SL lesions had a minimal effect on all radiological variables, except for Meary’s angle and HMA (Figure 5). The median accuracy of injury type predictions based on radiological outcomes was 41% (range 29–56).

Additional bony procedures were less commonly used for reconstruction surgery and varied substantially between Type A (70.1%), Type B (65.7%), and Type C (25.5%). They were also used significantly less in the post-traumatic group (Table 7).

## 4. Discussion

This study hypothesized that an SL injury causes morphological changes in the foot, which are smaller in post-traumatic cases due to the acute onset. Based on the results of surgical exploration, we confirmed the presence of three injury types, as proposed earlier [5]. Except for the TC angle, we found a difference between the injury and control groups for all radiographic measurements. The degree of radiographic changes was similar in injury Types A and B and more significant overall than in Type C. The incidence of trauma as a cause of an SL injury was 42%, with substantial variation between Type A (21%), Type B (43%), and Type C (72%). These numbers only include cases reported by the patients themselves. Assuming some patients may have initially sustained an injury but have since forgotten due to the delayed onset of symptoms, the incidence of trauma could be higher.

Although the intraoperative injury pattern did not differ between traumatic and non-traumatic patients, injuries to the distal PT tendon were more frequently associated with SL injuries in traumatic and younger patients. In a pronation-abduction trauma, the PT muscle, being a static muscle with a minimal excursion rate during foot movement, thereby making the muscle-tendon unit rigid, may be susceptible to injury [23]. Another explanation could be that hyperactivation of the PT muscle may occur and serve as a protective mechanism. The presence of an accessory bone that is firmly connected to the navicular may, in particular, expose the distal PT tendon to injury. The larger the accessory bone, the more likely it is that rotational forces will be present in addition to distraction forces, which disrupt the fibrous connection between the accessory bone and the navicular [5].

**Figure 6:**
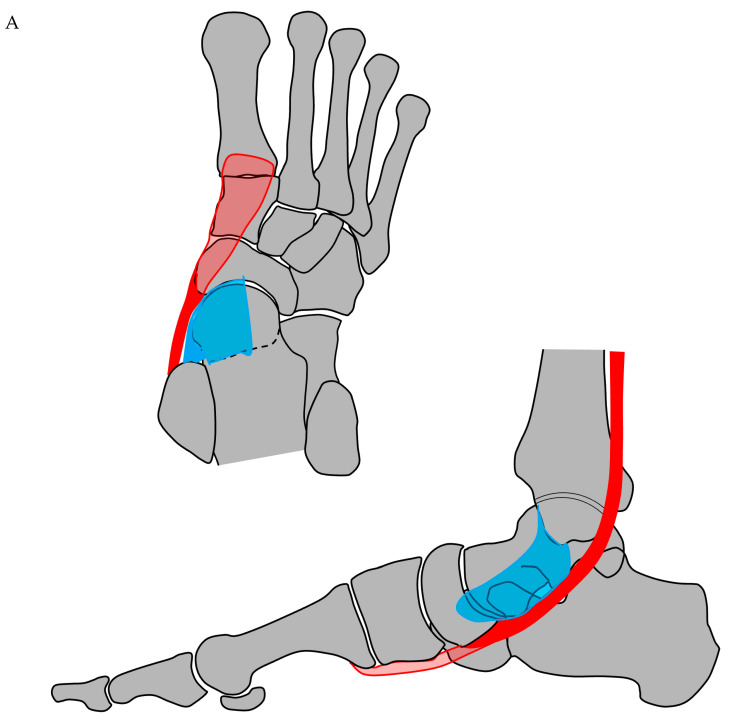

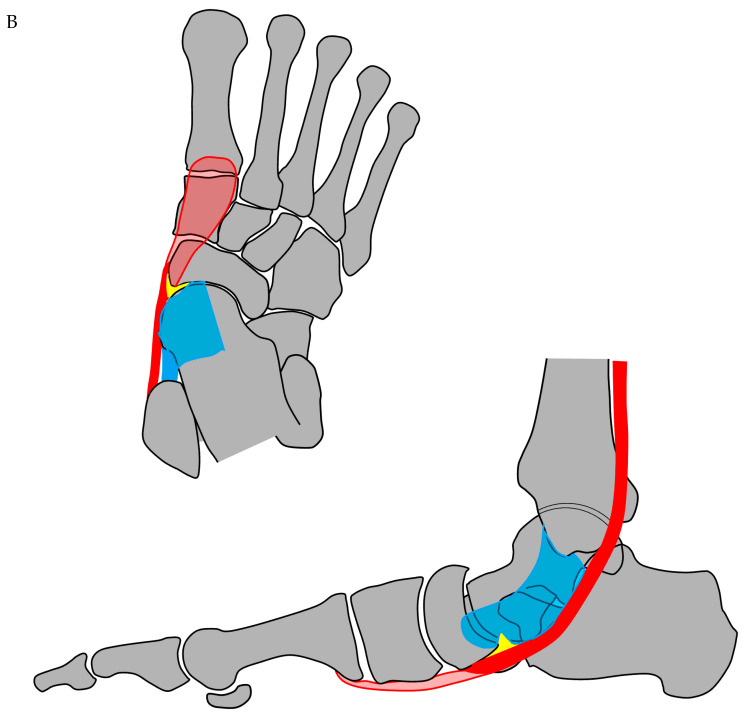
Drawing illustrating the insertion area of the central slip of the TP tendon on the tuberosity and along the medioplantar border of the navicular. (**A**) In a neutral position, the direction of the central slip aligns with the direction of the distal slips to the base of the 1st metatarsal and the 2nd to 4th metatarsals, respectively. (**B**) During a pronation-abduction trauma, the talar head becomes acutely plantarflexed and internally rotated, while the navicular is rotated externally. As the TP muscle is hyperactivated as a protective mechanism, the insertional fibers of its tendon at the navicular will be avulsed from proximal to distal, a process supported by the remaining intact slips at the base of the metatarsals that pull the PT tendon away from the navicular, resulting in an avulsion injury (yellow area). Typically, the SL will also be avulsed at the medioplantar aspect of the navicular.

In all cases, we found an isolated injury to the central slip of the PT tendon insertion at the navicular. In contrast, the connecting slips at the base of the metatarsals and the sustentaculum tali remained intact [3,24,25,26,27]. It may stem from the avulsion of insertional fibers at the navicular from proximal to distal when the talar head becomes acutely plantarflexed and internally rotated, while the navicular is rotated externally. This process is supported by the intact slips at the base of the metatarsal, which pull the PT tendon away from the navicular (Figure 6). This could explain why many patients, after sustaining such an injury, report feeling a whip or even a bang at the medial midfoot, and most patients demonstrate a relatively preserved function of the PT tendon once the acute pain has subsided. Consequently, the avulsion injury of the PT tendon may often be missed initially, becoming evident later due to the associated rupture of the SL, which leads to subsequent overload of the PT tendon.

A central question was how much the type of injury may determine foot deformity, allowing for predictions about the injury type based on radiologic findings. Except for the talocalcaneal angle, all measurements on standard weight-bearing radiographs differed between the injury group (or any single injury type) and the control group, indicating that injuries to the SL, with or without involvement of the PT tendon, result in flatfoot deformity. However, the average accuracy of predictions for injury type using radiographic measurements alone was only 42% (with an accuracy expected by chance being 33%) and could be slightly improved by including predictors such as sex, age, or trauma.

Not surprisingly, deformities were lower in post-traumatic cases than in those with insidious injury to the SL. Chronic overload of the deltoid-spring ligament complex may initially lead to its distension, followed by a subsequent partial to complete rupture of the SL [10]. In contrast, a rupture of the SL after acute trauma was found to have less impact on foot deformity, indicating that other ligamentous structures may be involved in supporting the talar head and protecting the foot against flatfoot deformity. Therefore, additional bony procedures were less commonly necessary for the following reconstruction surgery. This supports the hypothesis that the SL is not the only restraint, as the release of this ligament does not result in immediate deformity without cyclic loading [10,28,29]. Other ligamentous restraints may be affected by cyclic loading, causing deformity once the primary restraint has been lost. Biomechanical models that apply cyclic loading following the release of the SL have produced deformities similar to those seen in patients with PT tendon insufficiency [30,31,32].

Most recently, peritalar subluxation has been recognized as a crucial process in the development of progressive flatfoot deformity [33,34]. Weight-bearing radiographs were found to be inferior to weight-bearing CT in assessing peritalar subluxation [35]. As proposed by Benink [36], we used the talocalcaneal overlap as a tarsal index to indicate peritalar subluxation, as well as the internal rotation and plantarflexion of the talar head. We found it to be a reliable measure for describing the deformity resulting from an injury to the SL.

It has been widely recognized that a flatfoot deformity after an SL injury can result in compensatory varus deformity of the forefoot [37,38]. Several measurements were proposed to assess this forefoot deformity based on standard weight-bearing radiographs [37], but they mostly remained less reliable than measurements obtained using weight-bearing CT [35]. We have used the naviculo-cuboid overlap. Though we found this measure correlated with Meary’s angle, we could not validate its reliability. Nevertheless, the naviculo-cuboid overlap was, as the talocalcaneal overlap, lower in post-traumatic patients, indicating that an acute isolated SL injury does not cause an immediate forefoot varus deformity, suggesting that forefoot varus deformity is instead the result of chronic overload and subsequent incompetence of the SL.

An injury to the SL in its intermediate part at the junction with the tibiospring ligament was found to have the highest impact on foot deformity, particularly on Meary’s angle and the heel moment arm. Incompetence at this location leads to a loss of restraint on the talar head against internal rotation and plantarflexion when the foot becomes loaded [39]. This, in turn, may increase heel valgus and a subsequent increase in the heel moment arm.

One major limitation of the study is that measurements of the foot deformity were based solely on single-shot radiographs. Despite systematically instructing the patients to remain relaxed, activation of the PT muscle could not be completely excluded. Repeating the radiography one or two times, or utilizing additional weight-bearing CT, would have helped to detect tense cases. However, such attempts would significantly increase patient exposure to radiation and lack ethical justification. Another limitation may be the prolonged observation period. Increasing knowledge of this pathology may have influenced our decision-making process in assessing and classifying the injuries. However, based on previous work focusing on understanding this pathology, we recognized three injury types of the deltoid-spring ligament complex potentially associated with a distal PT lesion. Furthermore, the sample size was insufficient to justify models considering all explanatory variables of interest simultaneously. Thus, the analysis was divided into two parts to avoid overfitting.

## 5. Conclusions

In this case-controlled study comparing intraoperatively identified injury patterns to the SL with radiographic measurements, we could distinguish the three types of SL injuries in all cases: an isolated ligamentous injury (Type A), a ligamentous injury associated with a distal injury of the PT tendon (Type B), and a ligamentous injury associated with a bony avulsion of the PT tendon (Type C). While all but one of the traditionally used radiographic measurements for flatfoot deformity became pathological following an SL injury, they did not predict the injury pattern of the SL. The incidence of trauma as the origin was as high as 42%. We found, in post-traumatic cases, that the extent of foot deformity was lower than in cases with insidious SL injuries due to chronic overload. Further studies are needed to get more insight into the development of flatfoot deformity as a result of an SL injury.

## Figures and Tables

**Table 1:** Classification of distal spring ligament injuries.

	**Type A**	**Type B**	**Type C**
Spring ligament	rupture/avulsion	(partial) avulsion	(partial) tear
Posterior tibial tendon	intact	bony avulsion (with os tibial externum)	avulsion from navicular tuberosity

**Table 2:** Radiographic measurements ^(1)^.

Measurement	Abbreviation	Unit/Criteria	Normal (Interquartile Range for Control Group)	Figure
*DP-View (horizontal plane)*				
- Talonavicular coverage angle	TNC	degree	7–15 degrees	Figure 2A
- Talo-1st metatarsal angle	TM	degree	4–11 degrees	Figure 2A
*Lateral view (sagittal plane)*				
- Lateral talo-1st metatarsal angle (Meary)	MA	degree	4–9 degrees	Figure 2B
- Calcaneal inclination angle (Pitch)	CP	degree	19–24 degrees	Figure 2B
- Talo-calcaneal angle	TC	degree	44–51 degrees	Figure 2B
- Talocalcaneal overlap	TCO	mm	34–44%	Figure 2C
- Naviculocuboid overlap	NCO	mm	51–66%	Figure 2C
- Talonavicular step	TNS	mm	0 mm	Figure 2C
*Hindfoot alignment view*				
- Heel moment arm	HMA	mm	−6–5 mm	Figure 2D

^(1)^ The intra-rater reliability in terms of ICC was good for all measured parameters (between 0.785 [0.54–0.91]) and (0.848 [0.66–0.94]). A similar conclusion was reached for inter-rater reliability (between 0.851 [0.76–0.91] and 0.813 [0.69–0.89]).

**Table 3:** Recording of intraoperative findings.

Deltoid Ligament	Lesion/Tear	no lesion/intact
		partial or complete lesion
Spring ligament	Lesion/Tear	partial lesion
		complete lesion
	Location	intermediate
		distal

**Table 4:** Baseline characteristics of included patients with SL injury and the control group.

	Injury Group						Control Group	
	Overall	Rupture Type			Associated Variables			*p*-Value ^1^
		A	B	C				
N (%)	198 (100)	77 (39)	70 (35)	51 (26)	Name	Coefficients and CI based on AICc selection	30	-
Age (median, range)	57 (13–86)	60 (20–84)	60 (13–86)	38 (14–81)	Type	A: 14.6 (8.1–21.2) B: 14.8 (8.5–21.0) C: ref	51 (40–66)	0.299
					Trauma (Yes)	−7.7 (−13.0–−2.5)		
					Sex M	−0.9 (−5.8–4.0)		
Sex (females, %)	127 (64)	46 (60)	44 (63)	37 (73)	Type	A: 1.8 (0.8–3.8) B: 1.6 (0.7–3.4)	21 (70)	0.750
Trauma (N, %)	83 (42)	16 (21)	30 (43)	37 (73)	Type	A: 0.15 (0.06–0.35) B: 0.43 (0.18–0.99)	-	-
					Age	0.97 (0.95–0.99)		
					Sex M	1.2 (0.6–2.4)		

^1^ The *p*-values were adjusted using the Benjamini–Hochberg correction and refer to the comparison between the injury group (all three injury types combined) and the control group, using the Mann–Whitney test (Age) or Fisher’s exact tests (Sex, Trauma).

**Table 5:** Intraoperative findings.

	**Lesion/Location**	**Type A**		**Type B**		**Type C**		**Total**	
		**n**	**%**	**n**	**%**	**n**	**%**	**n**	**%**
**Patients (n)**		77	38.9	70	35.3	51	25.8	198	100.0
**DL lesion**	None/intact	30	39.0	52	74.3	47	92.2	129	65.2
	Partial/incomplete	45	58.4	18	25.7	4	7.8	67	33.8
	Transmural/complete	2	2.6	0	0.0	0	0.0	2	1.0
**SL lesion**	Partial/incomplete	38	49.4	42	60.0	24	47.1	104	52.5
	Transmural/complete	39	50.6	28	40.0	27	52.9	94	47.5
	Interligamentous	72	93.5	2	2.9	0	0.0	74	37.4
	Distal	5	6.5	68	97.1	51	100.0	124	62.6
**PT lesion**	None/intact	25	32.5	0	0.0	0	0.0	25	100.0
	Diseased/partial	48	62.3	0	0.0	0	0.0	48	24.2
	Avulsion without bone	4	5.2	70	100.0	0	0.0	74	37.4
	Bony avulsion	0	0.0	0	0.0	51	100.0	51	25.8

DL = deltoid ligament; SL = spring ligament; PT = posterior tibial.

**Table 6:** Difference between the injury groups and the control group ^(1)^.

Response Variable	Associated Variables Coefficients and CI	
	Name	
TNC angle	SL lesion	partial: ref complete: 1.6 (−1.2–4.3)
	SL lesion location	distal: ref intermediate: −1.5 (−4.4–1.5)
	DL lesion	no: ref yes: 0.9 (−2.0–3.8)
TM angle	-	-
MA angle	SL lesion	partial: ref complete: 0.6 (−2.0–3.2)
	SL lesion location	distal: ref intermediate: 1.7 (−1.0–4.5)
	DL lesion	no: ref yes: 1.7 (−1.1–4.4)
CP angle	SL lesion location	distal: ref intermediate: −0.9 (−2.6–0.7)
	DL lesion	no: ref yes: −1.0 (−2.7–0.6)
TC angle	SL lesion	partial: ref complete: 0.8 (−1.0–2.5)
	SL lesion location	distal: ref intermediate: 1.3 (−0.7–3.2)
	DL lesion	no: ref yes: 1.1 (−0.8–3.0)
TCO	SL lesion	partial: ref complete: 2.6 (−1.6–6.8)
	SL lesion location	distal: ref intermediate: 2.3 (−2.1–6.8)
	DL lesion	no: ref yes: 1.9 (−2.4–6.3)
NCO	SL lesion	partial: ref complete: 1.7 (−2.9–6.4)
	SL lesion location	distal: ref intermediate: 4.0 (−1.0–9.1)
	DL lesion	no: ref yes: 2.7 (−2.5–7.8)
TNS	SL lesion	partial: ref complete: 0.6 (0.0–1.3)
	SL lesion location	distal: ref intermediate: 0.8 (0.1–1.4)
HMA	SL lesion location	distal: ref intermediate: 3.5 (0.3–6.8)
	DL lesion	no: ref yes: 1.6 (−1.7–5.0)

^(1)^ Association between injury patterns as predictor variables ([SL lesions], SL location, and deltoid ligament lesion [DL lesion]) and radiological ankle measurements in 198 patients, as compared with the control group. TNC = talonavicular coverage; TM = talo-first metatarsal; MA = Meary’s angle; CP = calcaneal Pitch; TC = talocalcaneal; TCO = talocalcaneal overlap; NCO = naviculocuboid overlap; TNS = talonavicular step; HMA = heel moment arm.

**Table 7:** Bony procedures in patients with and without trauma in various types of SL injuries.

	Patients			Medial Sliding		Lateral Column		No Osteotomy	
	Total	No Trauma	Trauma	No Trauma	Trauma	No Trauma	Trauma	No Trauma	Trauma
	N (%)	N (%)	N (%)	N (%)	N (%)	N (%)	N (%)	N (%)	N (%)
Type A	77 (38.8)	61 (79.2)	16 (20.8)	15 (24.6)	1 (6.3)	35 (57.4)	3 (18.7)	11 (18.0)	12 (75.0)
Type B	70 (35.4)	40 (57.1)	30 (42.9)	7 (17.5)	2 (6.7)	24 (60.0)	9 (30.0)	9 (22.5)	19 (63.3)
Type C	51 (25.8)	14 (27.5)	37 (72.5)	2 (14.3)	0 (0.0)	8 (57.1)	3 (8.1)	4 (28.6)	34 (91.9)
Overall	198 (100.0)	115 (58.1)	83 (41.9)	24 (20.9)	3 (3.6)	67 (58.2)	15 (18.1)	24 (20.9)	65 (78.3)

## Data Availability

The original contributions presented in the study are included in the article, further inquiries can be directed to the corresponding authors.
